# Bacterial co-infection raises in-hospital mortality of COVID-19 patients: a retrospective study

**DOI:** 10.3389/fmicb.2023.1206476

**Published:** 2023-06-29

**Authors:** Kaican Zong, Wen Li, Yingya Fu, Sha Zhang, Yi Liu, Shiying Li

**Affiliations:** ^1^Department of Respiratory Medicine, The Seventh People’s Hospital of Chongqing, Affiliated Central Hospital of Chongqing University of Technology, Chongqing, China; ^2^Department of Infectious Diseases, Key Laboratory of Molecular Biology for Infectious Diseases (Ministry of Education), Institute for Viral Hepatitis, The Second Affiliated Hospital, Chongqing Medical University, Chongqing, China

**Keywords:** bacterial co-infection, COVID-19, pneumonia, mortality, risk factor

## Abstract

**Background:**

We aim to explore whether the bacterial co-infection with COVID-19 will raise the in-hospital mortality.

**Methods:**

COVID-19 patients’ information were collected for analysis in our retrospective study. Neutrophil count and procalcitonin (PCT) were used to estimate whether there was a suspected bacterial co-infection.

**Results:**

The main baselines between the suspected bacterial infection (SBI) and no evidence of bacterial infection (NBI) groups were no significant differences. In SBI group, patients required more therapies than NBI group. There was significantly higher in-hospital mortality (26% vs.9%, *P* < 0.001) between SBI and NBI groups in overall population. And in each subgroup based on pneumonia inflammation index (PII), it also showed higher in-hospital mortality of COVID-19 patients with bacterial co-infection. With logistic regression models, it showed that bacterial co-infection was associated with significantly higher in-hospital mortality in overall population (OR 1.694, 95% CI 1.179–2.434, *p* = 0.004) and mild subgroup (OR 2.374, 95% CI 1.249–4.514, *p* = 0.008). The rate of bacterial co-infection in overall population was 51%. At the same time, it showed a significantly higher rate of bacterial co-infection in critical subgroup than severe subgroup (63% vs. 49%, *p* = 0.003), and than that in moderate subgroup (63% vs. 48%, *p* = 0.002) based on clinical classification. It showed a significantly higher rates of bacterial co-infection in severe subgroup than moderate subgroup (66% vs. 49%, *p* = 0.001) based on PII. The result showed that the risk factor associated with significantly higher in-hospital mortality was PII (OR 1.018, 95%CI 1.012 to 1.024, *P* < 0.001) with logistic regression models.

**Interpretation:**

Bacterial co-infection estimated by Neutrophil count and procalcitonin significantly raises in-hospital mortality of COVID-19 patients in overall population in our study. Its impact is more significant in mild and moderate PII subgroups. PII based on CT imaging combined with neutrophil count and PCT is beneficial for accurate differentiation of bacterial co-infection of COVID-19.

## Introduction

In recent three years, people all over the world have been affected by the outbreak of COVID-19 (Bhatraju et al., 2020; Huang et al., 2020). Similar to other countries around the world, the COVID-19 pandemic is an arduous challenge for Chinese medical and health institutions (Yang et al., 2020). The COVID-19 pandemic has changed the fate of many people. As of Feb 20, 2023, 757 million people have been diagnosed for COVID-19, and unfortunately, over 6 million people have not survived from it (WHO, n.d.). It has always been a research hotspot what factors can lead to the death of COVID-19 and what measures may save the lives of infected individuals (Grasselli et al., 2020; Zhou et al., 2020). At present, the researches about bacterial co-infection of COVID-19 were quite different from each other (Hughes et al., 2020; Kreitmann et al., 2020; Rawson et al., 2020). So it was difficult to reach a consensus whether the bacterial co-infection increased the mortality of COVID-19 patients. In our study, we aim to explore whether the community bacterial co-infection with COVID-19 will raise the mortality. At the same time, to explore what are the risk factors of community bacterial co-infection with COVID-19. In our study, we used neutrophil count and Procalcitonin (PCT) to estimate whether there was a suspected bacterial co-infection. Neutrophil count and PCT has proven useful in the early diagnosis of lower respiratory tract infections of bacterial origin (Banerjee and Opal, 2017; Langford et al., 2020; Lingscheid et al., 2022). Van Berkel et al. (2020) thought that the respective cutoff values of PCT for bacterial infection was 0.5 ng/mL. Pink et al. (2021) pointed out that PCT measurement on admission and during the course of the disease in patients with COVID-19 may be helpful in identifying secondary bacterial infections and guiding the use of antibiotic therapy. And the cut-off value of PCT was 0.55 ng/mL (Pink et al., 2021). In the study of Ming DK et al., they found that the relatively low PCT (<0.5 ng/mL) concentration in the first 48 h of admission might suggest no community-acquired bacterial co-infection in patients presenting with COVID-19 (Ming et al., 2021). So in our study, if neutrophil count was abnormal, or PCT was higher than 0.5 ng/mL, they were considered bacterial co-infection. Our study may be beneficial for developing the correct antibacterial treatment strategy and avoiding the adverse consequences of delayed antibacterial treatment or antibiotic abuse.

## Patients and methods

Our retrospective study included 1,018 patients’ information. They were admitted to the Second Affiliated Hospital of Chongqing Medical University and the Seventh People’s Hospital of Chongqing from December 2022 to January 2023 due to COVID-19. For bacterial co-infection, the COVID-19 patients were grouped as suspected bacterial co-infection (SBI) and no evidence of bacterial co-infection (NBI). The patients were classified as the SBI group if the any one of (1) and (2) were met: (1) Neutrophil count was abnormal (normal range 1.8–6.3 × 10^9^/L), (2) PCT was higher than 0.5 ng/mL. Meanwhile, the information of gender, age, comorbidities, C-reactive protein (CRP), blood gas analysis and chest CT imaging were collected within 48 h after admission. More information about treatments and outcomes were collected too. Before collecting complete information, we established inclusion and exclusion criteria. Inclusion criteria: a) all the patients was diagnosed by a positive SARS-CoV-2 Real Time-Polymerase Chain Reaction (RT-PCR) for a confirmed diagnosis of COVID-19, b) CT imaging findings met the standard of viral pneumonia. Exclusion criteria: a) age < 18 years, b) patients during pregnancy and lactation, c) no chest CT imaging or no pneumonia was found by chest CT imaging. At present, COVID-19 patients were classified into four grades as follows: mild, moderate, severe and critical (China National Health Commission, n.d.). The standard was “Diagnosis and Treatment Program for Novel Coronavirus Pneumonia (tenth Edition)” published by the National Health Commission and National Administration of Traditional Chinese Medicine (China National Health Commission, n.d.). According to the standard, these patients met moderate, severe and critical grades. In addition, we also classified patients into four grades as follows: mild (0 < PII ≤ 25), moderate (25 < PII ≤ 50), severe (50 < PII ≤ 75) and critical (75 < PII ≤ 100) according to pneumonia inflation index (PII) which was based on CT imaging (Zhou et al., 2020). PII was calculated from each CT scan which was a ratio that calculated as 100% times one-fortieth (1/40) of the sum of the lesion distribution score, the lesion size score, and the consolidation score (Zhou et al., 2020). If the sum score exceeded 40, it was set to 40. And the PII calculation method was as follows (Zhou et al., 2020): (1) the lesion distribution score had a maximum of 20 points. For each pulmonary anatomy, 1 point was scored, (2) The lesion size score also had a maximum of 20 points. If pulmonary segment with lesions filling more than 50% of its volume, it attributed 1 point, (3) The consolidation score had a maximum of 20 points, and each pulmonary segment with large patch of consolidation scored 1 point. We would attempt to use PII to evaluate in-hospital mortality of COVID-19 patients and their bacterial co-infection. The study was approved by the Research Ethics Commission of the Second Affiliated Hospital of Chongqing Medical University.

### Statistical analysis

The statistical analysis was performed with SPSS 23.0. Measurement data conforming to normal distribution were represented by means±standard deviations (mean ± SD), and the difference between groups was compared by t test of two independent samples. The measurement data that did not conform to the normal distribution were expressed by medians and range (median ± IQR), and the difference between groups was compared by Mann–Whitney U test of two independent samples. The counting data was expressed as a percentage and the chi-square test was used to compare the differences between groups. It was considered statistically significant if *p*-values<0.05. We used the odds ratios (ORs) and 95% confidence interval (CIs) to estimate the association between bacterial co-infection and in-hospital mortality, and the association between bacterial co-infection and its risk factors with multivariable adjusted logistic regression models.

## Results

### Baseline characteristics

A total of 1,018 patients with a diagnosis of COVID-19 pneumonia were evaluated. The baselines of the SBI and NBI groups were shown in Table 1. Between SBI and NBI groups, there were no significant differences in mean age (69.59 vs.70.51, *p* = 0.329), gender (percentage of male was 61% vs.58%, *p* = 0.251), frequency of chronic pulmonary disease (14% vs.18%, *p* = 0.107), chronic liver disease (4% vs.5%, *p* = 0.555), platelet count (164 vs.164, *p* = 0.997). In SBI group, frequency of diabetes (31% vs.25%, *p* = 0.042), cardiovascular diseases (50% vs.41%, *p* = 0.008), chronic kidney disease (21% vs.6%, *p* < 0.001) were higher than NBI group. It showed a significantly higher in neutrophil count (7.09 vs.3.71, *p* < 0.001), CRP (34.6 vs.12, *p* < 0.001), PCT (0.59 vs.0.1, *p* < 0.001), D-dimer (1.27 vs.0.76, *p* < 0.001), and significantly lower in oxygenation index (294.31 vs. 315.09, *p* = 0.017), lymphocyte count (0.88 vs.1.0, *p* = 0.002) between SBI and NBI groups. In SBI group, patients required more therapies than NBI group, such as systemic corticosteroids (47% vs.40%, *p* = 0.023), budesonide (20% vs.26%, *p* = 0.014), supplemental oxygen (74% vs.59%, *p* < 0.001), NIV (28% vs.18%, *p* < 0.001), IMV (14% vs.3%, *p* < 0.001), Azvudine (18% vs.15%, *p* = 0.269) and Paxlovid (18% vs.15%, *p* = 0.239).

**Table 1:** The baseline of COVID-19 patients between SBI and NBI group.

	SBI (*n* = 519)	NBI (*n* = 499)	*p*-value
Age, years (mean ± SD)	69.59 ± 15.27	70.51 ± 14.95	*p* = 0.329
Gender, male	317 (61%)	287 (58%)	*p* = 0.251
Diabetes	159 (31%)	124 (25%)	*p* = 0.042
Cardiovascular diseases	258 (50%)	206 (41%)	*p* = 0.008
Chronic pulmonary disease	75 (14%)	91 (18%)	*p* = 0.107
Chronic kidney disease	109 (21%)	29 (6%)	*p*<0.001
Chronic liver disease	22 (4%)	26 (5%)	*p* = 0.555
Laboratory tests			
Neutrophil count(median ± IQR) × 10^9^	7.09 (4.78–9.60)	3.71 (2.82–4.73)	*p*<0.001
Lymphocyte count(mean ± SD) × 10^9^	0.88 ± 0.65	1.00 ± 0.58	*P* = 0.002
Platelet count (median ± IQR) × 10^9^	164 (119–231)	164 (125–218)	*p* = 0.997
CRP (median ± IQR) – mg/dL	34.60 (6.97–94.62)	12 (3.12–44.19)	*p*<0.001
PCT (median ± IQR) – ng/mL	0.59 (0.12–2.54)	0.1 (0.06–0.16)	*p*<0.001
D-dimer (median ± IQR) – ng/mL	1.27 (0.62–3.20)	0.76 (0.39–1.33)	*p*<0.001
Oxygenation index (mean ± SD)	294.31 ± 143.10	315.09 ± 133.54	*p* = 0.017
Treatment received in hospital			
systemic corticosteroids	246 (47%)	201 (40%)	*p* = 0.023
budesonide	102 (20%)	131 (26%)	*p* = 0.014
supplemental oxygen	383 (74%)	294 (59%)	*p*<0.001
NIV	145 (28%)	92 (18%)	*p*<0.001
IMV	75 (14%)	16 (3%)	*p*<0.001
Azvudine	91 (18%)	74 (15%)	*p* = 0.269
Paxlovid	94 (18%)	76 (15%)	*p* = 0.239
Clinical type, *N*%			
Moderate	180 (35%)	194 (39%)	*p* = 0.172
Severe	245 (47%)	253 (51%)	*p* = 0.363
Critical	90 (17%)	52 (10%)	*p* = 0.002
PII, *N*%			
Mild	238 (46%)	293 (59%)	*p* = 0.001
Moderate	160 (31%)	164 (33%)	*p* = 0.501
Severe	89 (17%)	35 (7%)	*p*<0.001
Critical	32 (6%)	7 (1%)	*p*<0.001
Outcomes			
hospitalization days(mean ± SD)	8.85 ± 5.69	8.90 ± 5.50	*p* = 0.886
in-hospital mortality	135 (26%)	45 (9%)	*p*<0.001

SD, standard deviation; IQR, interquartile rang; SBI, suspected bacterial infection; NBI, no evidence of bacterial infection; PII, pneumonia inflammation index; CRP, C-reactive protein; PCT, procalcitonin; NIV, non-invasive mechanical ventilation; IMV, invasive mechanical ventilation.

### Primary outcomes: in-hospital mortality

**Figure 1:**
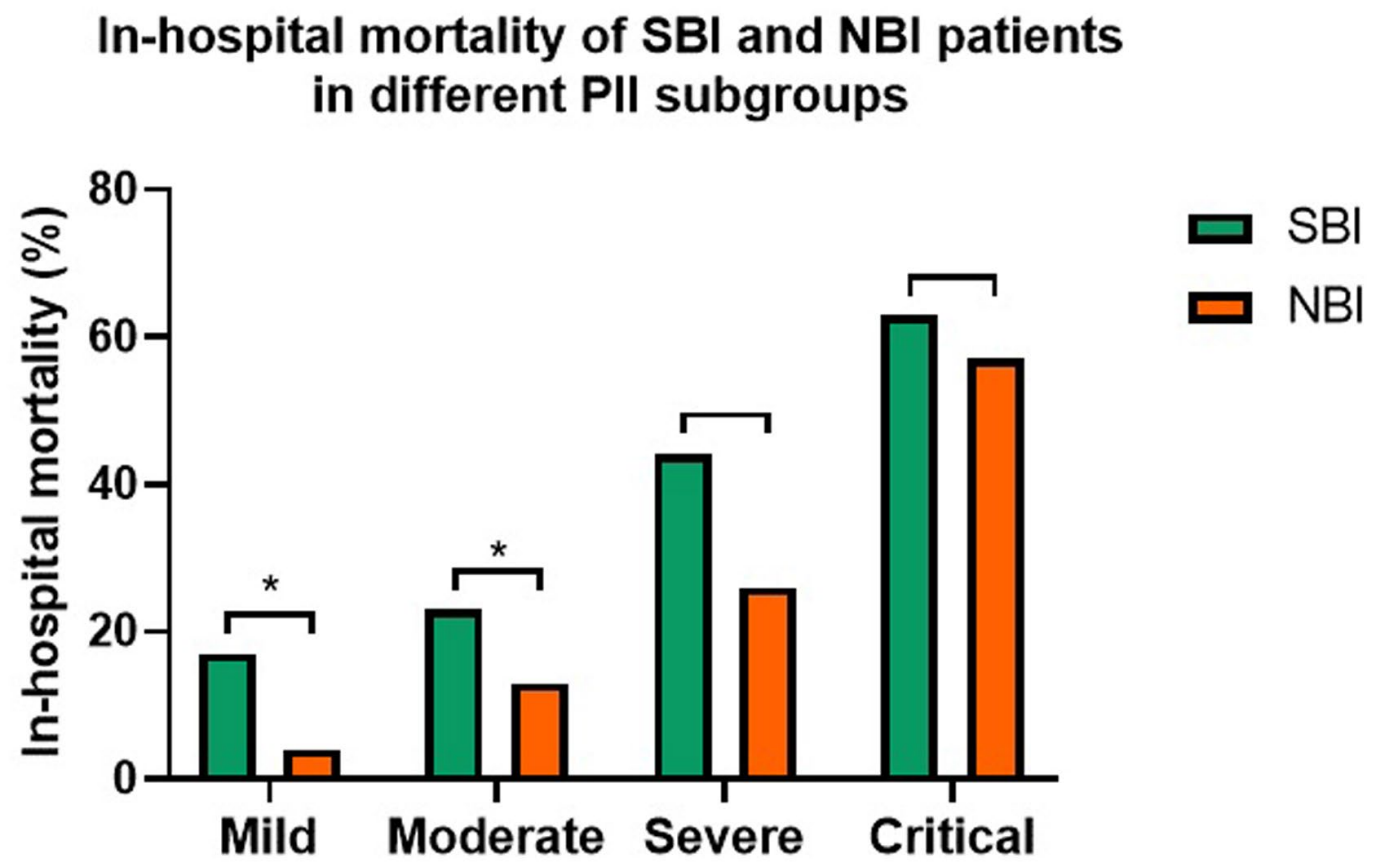
The in-hospital mortality of COVID-19 patients in different PII subgroups with bacterial co-infection or not (*p < 0.05).

There was significantly higher in-hospital mortality (26% vs.9%, *p* < 0.001) between SBI and NBI groups in overall population (Table 1). And in each subgroup based on PII (Figure 1), it also showed higher in-hospital mortality of COVID-19 patients with bacterial co-infection, although no significant differences in severe subgroup (44% vs. 26%, *p* = 0.069) and critical subgroup (63% vs. 57%, *p* = 1.000). But in-hospital mortality was significantly higher in mild subgroup (17% vs. 4%, *p* < 0.001) and moderate subgroup (23% vs. 13%, *p* = 0.028).

Further more, we analyzed the association of bacterial co-infection and in-hospital mortality of COVID-19 patients with logistic regression models (Table 2). The result showed that bacterial co-infection was associated with significantly higher in-hospital mortality in overall population (OR 1.679, 95% CI 1.202–2.344, *p* = 0.002), mild subgroup (OR 5.179, 95% CI 2.594–10.342, *p* < 0.001) and moderate subgroup (OR 1.977, 95% CI 1.096–3.565, *p* = 0.023) with univariate analysis, but no significant association in severe subgroup (OR 2.253, 95% CI 0.948–5.357, *p* = 0.066) and critical subgroup (OR 1.25, 95% CI 0.238–6.569, *p* = 0.792). With multivariable adjusted logistic regression models, it showed that bacterial co-infection was associated with significantly higher in-hospital mortality in overall population (OR 1.694, 95% CI 1.179–2.434, *p* = 0.004) and mild subgroup (OR 2.374, 95% CI 1.249–4.514, *p* = 0.008), but no significant association in moderate subgroup (OR 0.842, 95%CI 0.446 to 1.592, *p* = 0.598), severe subgroup (OR 1.309, 95% CI 0.484–3.54, *p* = 0.595) and critical subgroup (OR 0.678, 95% CI 0.16–2.87, *p* = 0.598).

**Table 2:** Association of bacterial co-infection and in-hospital mortality of COVID-19 patients in overall and PII subgroups.

In-hospital mortality	Univariate analysis				Multivariate analysis			
	OR	Lower.95	Upper.95	*p*-value	OR	Lower.95	Upper.95	*p*-value
Overall (*n* = 1,018)	1.679	1.202	2.344	*p* = 0.002	1.694	1.179	2.434	*p* = 0.004
Mild subgroup (*n* = 374)	5.179	2.594	10.342	*p* = 0.000	2.374	1.249	4.514	*p* = 0.008
Moderate subgroup (*n* = 324)	1.977	1.096	3.565	*p* = 0.023	0.842	0.446	1.592	*p* = 0.598
Severe subgroup (*n* = 124)	2.253	0.948	5.357	*p* = 0.066	1.309	0.484	3.54	*p* = 0.595
Critical subgroup (*n* = 39)	1.25	0.238	6.569	*p* = 0.792	0.678	0.16	2.87	*p* = 0.598

PII, pneumonia inflammation index; OR, odds ratio; CI, confidence interval.

### Rate of bacterial co-infection

**Figure 2:**
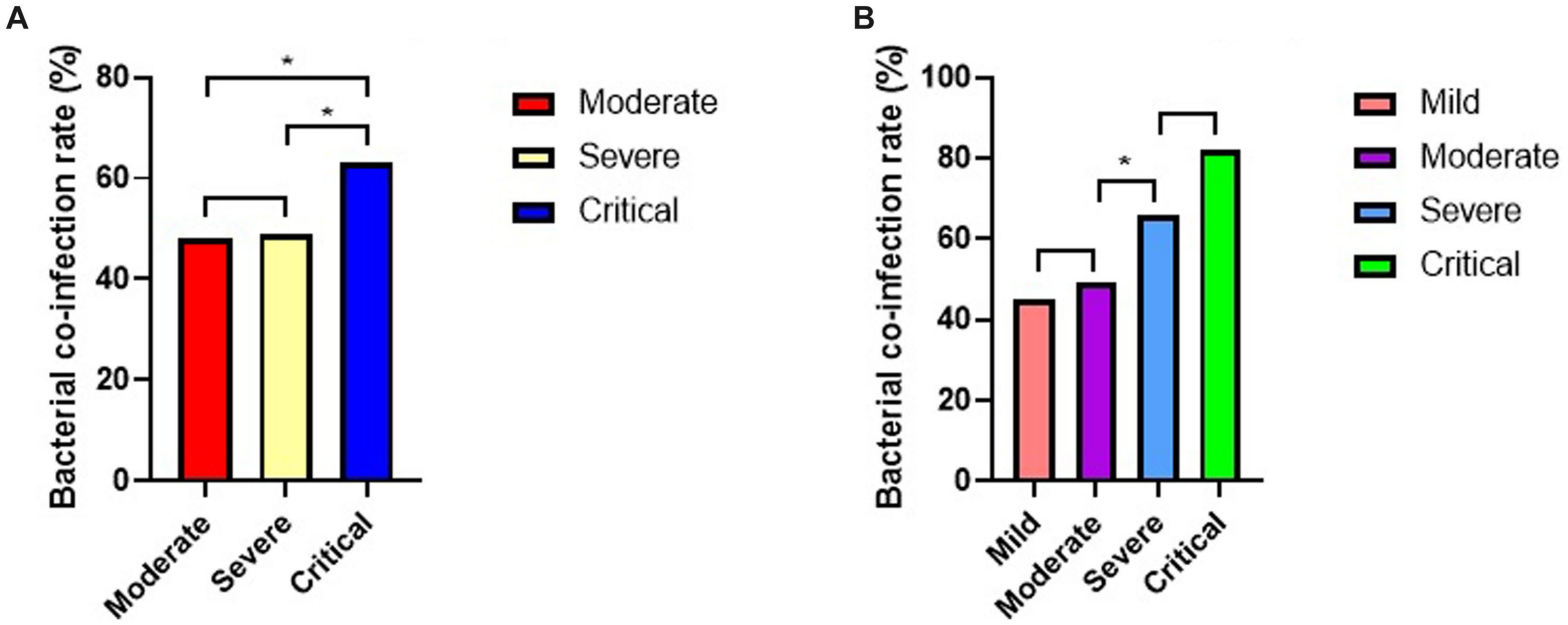
Rates of bacterial co-infection of COVID-19 patients in different subgroups (*p < 0.05). **(A)** Rates of bacterial co-infection in different clinical subgroups. **(B)** Rate of bacterial co-infection in different PII subgroups.

The rate of bacterial co-infection in overall population was 51%. At the same time, we analyzed the rates of bacterial co-infection of COVID-19 patients in different subgroups (Figure 2). Based on clinical classification (Figure 2A), the result showed a significantly higher rate of bacterial co-infection in critical subgroup than severe subgroup (63% vs. 49%, *p* = 0.003), and than that in moderate subgroup (63% vs. 48%, *p* = 0.002), but no significant difference between moderate and severe subgroups (49% vs. 48%, *p* = 0.784).

Based on PII (Figure 2B), there were no significant differences in rates of bacterial co-infection between critical and severe subgroups (82% vs. 66%, *p* = 0.075), also between moderate and mild subgroups (49% vs. 45%, *p* = 0.204). But it showed a significantly higher rates of bacterial co-infection in severe subgroup than moderate subgroup (66% vs. 49%, *p* = 0.001).

### Factors related to bacterial co-infection

Using multivariable adjusted logistic regression models (Table 3), the result showed that the risk factor associated with significantly higher in-hospital mortality was PII (OR 1.018, 95%CI 1.012 to 1.024, *p* < 0.001). While all of older age (OR 1.003, 95%CI 0.994 to 1.013, *p* = 0.497), gender (OR 1.034, 95%CI 0.793 to 1.348, *p* = 0.805), with diabetes (OR 0.77, 95%CI 0.575 to 1.032, *p* = 0.081), cardiovascular diseases (OR 1.145, 95%CI 0.865 to 1.514, *p* = 0.344), chronic pulmonary disease (OR 0.94, 95%CI 0.663 to 1.332, *p* = 0.727), chronic kidney disease (OR 1.061, 95%CI 0.721 to 1.561, *p* = 0.765), and chronic liver disease (OR 0.957, 95%CI 0.535 to 1.709, *p* = 0.881) were not significantly associated with higher in-hospital mortality.

**Table 3:** Factors related to bacterial co-infection of COVID-19 patients with logistic regression model.

Variables	OR	Lower.95	Upper.95	*p*-value
PII	1.018	1.012	1.024	*p*<0.001
Age	1.003	0.994	1.013	*p* = 0.497
Gender	1.034	0.793	1.348	*p* = 0.805
Diabetes	0.77	0.575	1.032	*p* = 0.081
Cardiovascular diseases	1.145	0.865	1.514	*p* = 0.344
Chronic pulmonary disease	0.94	0.663	1.332	*p* = 0.727
Chronic kidney disease	1.061	0.721	1.561	*p* = 0.765
Chronic liver disease	0.957	0.535	1.709	*p* = 0.881

OR, odds ratio; PII, pneumonia inflammation index.

## Discussion

Many studies have focused on bacterial co-infection of COVID-19, but the reported rates varied greatly between different studies (Hughes et al., 2020; Kreitmann et al., 2020; Rawson et al., 2020). Some studies pointed out that the proportion of bacterial co-infection in COVID-19 patients was very low (Lansbury et al., 2020; Garcia-Vidal et al., 2021). But contrary to this finding, the use of antibiotics was very high, although there was no evidence of bacterial co-infection (Lansbury et al., 2020; Rawson et al., 2020). This contradiction reminded us that clinicians might not have reliable standards to guide them in antibacterial treatment. And it also reminded us that it might be not appropriate to guide antibacterial strategies based on bacteriology evidence from patients’ clinical samples. In our study, neutrophil count and PCT level were used to distinguish the possibility of bacterial co-infection of COVID-19. The results showed that there were no significant differences in demographic characteristics between SBI group and NBI group. However, the indicators such as neutrophil count, PCT and CRP related to bacterial infection in the SBI group were significantly higher than those in the NBI group. At the same time, patients in the SBI group might be more seriously because of more treatment support required. More importantly, in the overall population, the in-hospital mortality of COVID-19 in the SBI group was significantly higher than that in the NBI group. Therefore, our study represented that antibacterial strategies based on this way to distinguish bacterial co-infection of COVID-19 may contribute to modify in-hospital mortality.

Further more, we analyzed the in-hospital mortality of COVID-19 in different PII subgroups with bacterial co-infection or not. The results showed that the in-hospital mortality of the four subgroups based on PII were as follows: critical subgroup (63% vs. 57%), severe subgroup (44% vs. 26%), moderate subgroup (23% vs. 13%), mild subgroup (17% vs. 4%) with bacterial co-infection or not. This result indicated that bacterial co-infection significantly raised in-hospital mortality in mild and moderate subgroups classified by PII, while in severe and critical subgroups, bacterial co-infection did not significantly affect in-hospital mortality of COVID-19. It also found that bacterial co-infection was an independent risk factor for in-hospital mortality in the overall population and mild subgroup with multivariable adjusted logistic regression models. Therefore, it was beneficial for predicting the correlation between bacterial co-infection and in-hospital mortality of COVID-19 patients classified by PII which based on CT imaging within 48 h of admission. Similar to our results, Peng S et al. pointed out that the PII correlated well with the clinical classifcation, and PII could be used to monitor the outcome of COVID-19 pneumonia (Peng et al., 2021).

Arentz et al. (2020) considered that the rate of bacterial co-infection of COVID-19 was 4.8%, and Chen et al. (2020) pointed out that the rate of bacterial co-infection of COVID-19 was 1%. In their study, they both found the rates of bacterial co-infection of COVID-19 was low, but it could not explain the high rates of receiving antibiotic. In our study, the overall rate of bacterial co-infection of COVID-19 was 51%, although it was still lower than the rate of receiving antibiotic. It may be closer to the actual situation and the rate of using antibiotics. In addition, we analyzed the rates of bacterial co-infection of COVID-19 patients in different subgroups. Based on clinical classification, the result showed a significantly higher rate of bacterial co-infection in critical subgroup than severe and moderate subgroup. Based on PII, rates of bacterial co-infection between critical and severe subgroups were no significant differences, also between moderate and mild subgroups. But the rate of bacterial co-infection in severe subgroup was significantly higher than moderate subgroup. This result indicated that there was a better correlation between the bacterial co-infection and the severity of COVID-19 patients based on PII.

Moreno-García et al. (2022) pointed out in their study, the independent risk factors for co-infection of COVID-19 were oxygen saturation ≤ 94%, ferritin levels<338 ng/mL and PCT higher than 0.2 ng/mL. In our study, we classified the suspected bacterial co-infection of COVID-19 if PCT was higher than 0.5 ng/mL. Finally, we found that there was no correlation between the bacterial co-infection and age, gender and comorbidities, while significant correlation with PII. This result indicated that PII was an independent risk factor of bacterial co-infection of COVID-19.

Our study only analyzed the impact of community bacterial co-infection on in-hospital mortality, but payed no attention to the hospital acquired bacterial co-infection. Furthermore, we did not analyze the antimicrobial treatment during hospitalization, because doctors tended to use antibiotics even without evidence of bacterial infection.

In conclusion, bacterial co-infection estimated by Neutrophil count and procalcitonin significantly raises in-hospital mortality of COVID-19 patients in overall population in our study. Its impact is more significant in mild and moderate PII subgroups. PII is an independent risk factor of bacterial co-infection of COVID-19. CT imaging combined with neutrophil count and PCT is beneficial for accurate differentiation of bacterial co-infection of COVID-19.

## Data availability statement

The original contributions presented in the study are included in the article/supplementary material, further inquiries can be directed to the corresponding author.

## Ethics statement

The studies involving human participants were reviewed and approved by the Research Ethics Commission of the Second Affiliated Hospital of Chongqing Medical University. Written informed consent for participation was not required for this study in accordance with the national legislation and the institutional requirements.

## Author contributions

SL and KZ designed the study and analyzed the data. All authors contributed to the article and approved the submitted version.

## Funding

This work was supported by the General Program of Chongqing Natural Science Foundation (CSTB2022NSCQ-MSX0901) and Kuanren Talents Program of the Second Affiliated Hospital of Chongqing Medical University (kryc-yq-2204).

## Conflict of interest

The authors declare that the research was conducted in the absence of any commercial or financial relationships that could be construed as a potential conflict of interest.

## Publisher’s note

All claims expressed in this article are solely those of the authors and do not necessarily represent those of their affiliated organizations, or those of the publisher, the editors and the reviewers. Any product that may be evaluated in this article, or claim that may be made by its manufacturer, is not guaranteed or endorsed by the publisher.
